# The transition to universal health coverage in low and middle-income countries: new opportunities for community pharmacists

**DOI:** 10.1186/s40545-020-00213-7

**Published:** 2020-05-26

**Authors:** Vivian Naidoo, Fatima Suleman, Varsha Bangalee

**Affiliations:** Discipline of Pharmaceutical Sciences, University of Kwa-Zulu Natal, F-Block, Room F2-519, Westville Campus, Durban, South Africa

**Keywords:** Universal health coverage, Pharmacist, Low and middle-income countries

The transition towards universal health coverage (UHC) in several low and middle-income countries (LMICs) has been with the intent of improving accessibility and affordability of healthcare [1]. South Africa is one example of an LMIC that has been progressively moving towards the implementation of a National Health Insurance (NHI). The NHI aims to provide a comprehensive, integrated, and accessible universal tax-funded system across all socio-economic groups in South Africa [2]. The NHI was first proposed in 1994; but it was only between 2012 and 2017 that the implementation phase of the NHI commenced. In August 2019, the NHI bill was introduced to parliament and is currently under consideration by the National Assembly of South Africa’s portfolio committee [3]. The successful implementation of the NHI requires widespread collaboration as well as a complete reassessment of roles and practices of pharmacists, to better align with the needs of UHC [4]. Using South Africa as an example, this article aims to highlight some of the underutilized services that community pharmacists in LMICs could provide to aid in the successful implementation of UHC.

As part of the NHI policy implementation the government has outlined the re-engineered of primary health care (PHC) services and an enhanced referral system to improve accessibility to quality health services. The proposed change in pharmacist scope of practice aims at spearheading the re-engineering by focusing their skills on PHC in an attempt to reduce the influx into higher levels of care [5]. Community pharmacists are ideally placed, as they are most often the first point of call for patients seeking medical advice [6]. Currently, there are 15,924 qualified registered pharmacist in South Africa servicing an estimated population of 58,8 million people [7, 8]. There are 3436 community pharmacies, 620 public hospitals and 311 private hospitals in South Africa [8]. Their knowledge and skill set, accessibility, and extended trade hours further reflects the capacity of the profession to contribute toward enhancing and complementing access and primary healthcare coverage under UHC [9, 10]. Increasing the involvement of pharmacists at a primary health care level would consequentially benefit other public sector establishments by relieving the workload and pressure on staff at these facilities [11], especially for patients that have been stabilized for treatment of chronic non-communicable diseases.

Pharmacies are multi-product, multi-service facilities that are essential to a health care system. The past few decades, in particular, has seen the pharmacy profession in LMICs aim to transition from a distributive medication focus to a patient-centred focus. This is, in part, based on the premise that providing medicines alone is not sufficient to achieve treatment goals, achieve efficiency, or provide cost-effective solutions [12, 13]. In contrast to many high-income countries, pharmacists are an underutilized resource for patient care in LMICs [14]. Inadequate reimbursement policies, health system structuring and legislature are some of the contributing factors of this [9, 15].

The practice of pharmacy in South Africa is set to change; with the aim of introducing skills that have been previously underutilized by this group of health professionals. According to the Pharmaceutical Society of South Africa (PSSA), a pharmacists scope of practice also encompasses immunization, selective reproductive health services, screening and monitoring in a PHC setting and provision of information [15]. Some of these services are however, only offered at select pharmacies as currently in South Africa further training, and certification is required before a pharmacist is allowed to offer them. Therefore if pharmacists are to be integrated with the broader primary health care system, greater strides in improving the training and early certification for providing these services at an undergraduate level would be beneficial [5].

The increasing incidence and prevalence of chronic disease, increasingly complex range of medicinal uses and poor adherence to prescribed medication have provided additional opportunities for pharmacists to deliver patient targeted services, creating a higher demand for interdisciplinary, team-based approaches to deliver these services [16]. This presents the opportunity for the practice of collaborative drug therapy management (CDTM). CDTM is an agreement between one or more physicians and a pharmacist under a protocol whereby the physician makes the diagnosis, supervises patient care, and refers the patient to the pharmacist for performing services such as patient assessments; ordering drug-therapy-related laboratory tests; administering drugs; and selecting, initiating, monitoring, continuing, and adjusting drug regimens [17]. The challenges of implementing this system rests with current restrictive regulations such as the Medicines and Related substances Act (101 of 1965 as amended), which currently prohibits doctors and pharmacists from operating together [18]. Additionally outdated compensation schemes from government and medical insurers must be transformed to allow for fair reimbursement of expanded pharmaceutical care in CDTM establishments [16].

Two advanced pharmacist-initiated services that may run in parallel to CDTM are the New Medicine Service (NMS) and Medicine Treatment Management (MTM) service. While these services are independent of the provision of medicines they may run in conjunction with daily procedures, as they target both health related issues and the prevention of medication-related morbidity and mortality [12, 19]. They include reviewing medication regimens, the provision of personal medication records, the development of a medication-related action plan (which may include therapeutic recommendations, a provider referral), documentation, monitoring, and follow-up as required [11]. The benefits of this collaboration would improve patient adherence and rational prescribing of medicines by practitioners, as pharmacists would compile and maintain information on a patient’s medical history, mainly newly introduced medicines, and provide advice to all healthcare professionals, as necessary [12, 19]. In order to promote advanced services under UHC, systems need to be developed to ensure that community pharmacists have access to patient/electronic health records. Unlike hospital pharmacists, community pharmacists have inadequate access to the diagnosis, medical history, laboratory values, and the drug history of their patients, information that is critical to better-utilizing pharmacists [13].

The provision of advanced services additionally enables pharmacists to identify health conditions prominent in the community and initiate health promotion campaigns. From a public health standpoint, community pharmacies can offer services across a range of relevant, previously underutilized areas of health needs including test and treat interventions for HIV positive patients [20]. It is anticipated that community pharmacies could be integrated into the referral pathway, whereby pharmacists can perform interventions or act as the gatekeepers for referrals to a local clinic/GP where a more advanced intervention is required. Furthermore, pharmacist will also play a vital part in the downward referral system by means of the CCMDD (Chronic Central Medicine Dispensing Distribution) program; whereby patients are decentralized to points close to their community from higher health tiers based on the stable management of chronic conditions.

There are also roles from a health system perspective that pharmacists can excel in, such as medicines supply management (in order to prevent frequent stock outs and loss of stock) and health technology assessment. This will increasingly become important with the use of ATMs for dispensing medicines as well as drones for the remote delivery and supply of medicines. UHC affords pharmacists the opportunity to evolve in their roles and become more engaged at community and system levels.

## Conclusion

While previously unexplored opportunities for pharmacists to provide advanced patient care services emerge in South Africa, several considerations need to be addressed. Firstly, a change in attitude regarding the pharmacy profession by those practicing it, by patients utilizing the services and by other health professionals collaborating in the future of shared patient care. Other considerations include addressing training gaps, infrastructure requirements, changes to legislation and finally remuneration packages.

## Data Availability

Not Applicable.

## Abbreviations

LMICs: Low and middle-income countries
NHI: National Health Insurance
UHC: Universal Health Coverage
DOH: Department of Health
SAPC: South African Pharmacy Council
MTM: Medicine and Therapy Management
NMS: New Medicine Services
CDTM: Collaborative Drug Therapy Management
CCMDD: Chronic Central Medicine Dispensing and Distribution
